# The effect of an educational intervention on coronary artery bypass graft surgery patients' participation rate in cardiac rehabilitation programs: a controlled health care trial

**DOI:** 10.1186/1471-2261-11-60

**Published:** 2011-10-08

**Authors:** Rachel Dankner, Galit Geulayov, Arnona Ziv, Ilia Novikov, Uri Goldbourt, Yaakov Drory

**Affiliations:** 1 Unit for Cardiovascular Epidemiology, the Gertner Institute for Epidemiology and Health Policy Research, Sheba Medical Center, Tel Hashomer 52621, Israel; 2 Department for Data Management, the Gertner Institute for Epidemiology and Health Policy Research, Sheba Medical Center, Tel Hashomer 52621, Israel; 3 Unit for Biostatistics, the Gertner Institute for Epidemiology and Health Policy Research, Sheba Medical Center, Tel Hashomer 52621, Israel; 4 Division of Epidemiology and Preventive Medicine, School of Public Health, Sackler Faculty of Medicine, Tel Aviv University, Ramat Aviv, Israel; 5 Sackler Faculty of Medicine, Tel Aviv University, Ramat Aviv, Israel

**Keywords:** coronary artery disease, educational program, patient rights, tertiary prevention, intervention

## Abstract

**Background:**

Cardiac rehabilitation has a beneficial effect on the prognosis and quality of life of cardiac patients, and has been found to be cost-effective. This report describes a comprehensive and low cost educational intervention designed to increase the attendance at cardiac rehabilitation programs of patients who have undergone coronary artery bypass graft surgery.

**Methods/Design:**

A controlled prospective intervention trial. The control arm comprised 520 patients who underwent coronary artery bypass graft surgery between January 2004 and May 2005 in five medical centers across Israel. This group received no additional treatment beyond usual care. The intervention arm comprised 504 patients recruited from the same cardiothoracic departments between June 2005 and November 2006. This group received oral and written explanations about the advantages of participating in cardiac rehabilitation programs and a telephone call two weeks after hospital discharge intended to further encourage their enrollment. The medical staff attended a one-hour seminar on cardiac rehabilitation. In addition, it was recommended that referral to cardiac rehabilitation be added to the letter of discharge from the hospital. Both study groups were interviewed before surgery and one-year post surgery. A one-year post-operative interview assessed factors affecting patient attendance at cardiac rehabilitation programs, as well as the structure and content of the cardiac rehabilitation programs attended. Anthropometric parameters were measured at pre- and post-operative interviews;- and medical information was obtained from patient medical records. The effect of cardiac rehabilitation on one- and three-year mortality was assessed.

**Discussion:**

We report a low cost yet comprehensive intervention designed to increase cardiac rehabilitation participation by raising both patient and medical staff awareness to the potential benefits of cardiac rehabilitation.

**Trial registration:**

ClinicalTrials.gov: NCT00356863

## Background

When we initiated this interventional study in 2004, the effectiveness of cardiac rehabilitation (CR) programs for promoting health, psychological status and functioning; and for decreasing symptoms of angina pectoris and heart failure, in post-coronary artery bypass graft (CABG) patients had already been well established [1-5]. Though shown to be highly cost effective [6,7], CR programs reported low participation rates [8]. Low referral rates and low patient motivation, both of which were due to lack of knowledge regarding potential health benefits, were identified as the main barriers to cardiac rehabilitation [9,10]. Simple interventions, such as two post-CABG telephone calls [11] and more comprehensive CR programs [12] demonstrated positive effect.

We designed a simple intervention aimed to increase participation of post-CABG surgery patients in CR programs, by increasing patient and staff awareness of the benefits, availability, and patient eligibility to participate in these programs.

## Methods/Design

### Study initiation

#### Aim

The overall aim was to assess the effect of an easy-to-implement intervention on participation of post-CABG patients in CR programs.

#### Specific Objectives

1. To examine the effect of a hospital-based intervention program on the rate of attendance of post-CABG patients at CR programs;

2. To investigate the effect of sociodemographic factors on CR participation;

3. To assess the effect of CR participation on medical as well as psychological outcomes of post-CABG patients, and on their quality of life.

#### Hypotheses

1. We hypothesized that raising the awareness of the potential health benefits of CR among medical staff and CABG patients would significantly increase patient attendance at CR programs;

2. We hypothesized that patients who participated in CR would show greater improvements in medical outcomes and quality of life, reduced levels of anxiety and depression, and increased 1 and 3-year survival rates, compared to patients who did not participate in CR.

#### Design

**Figure 1:**
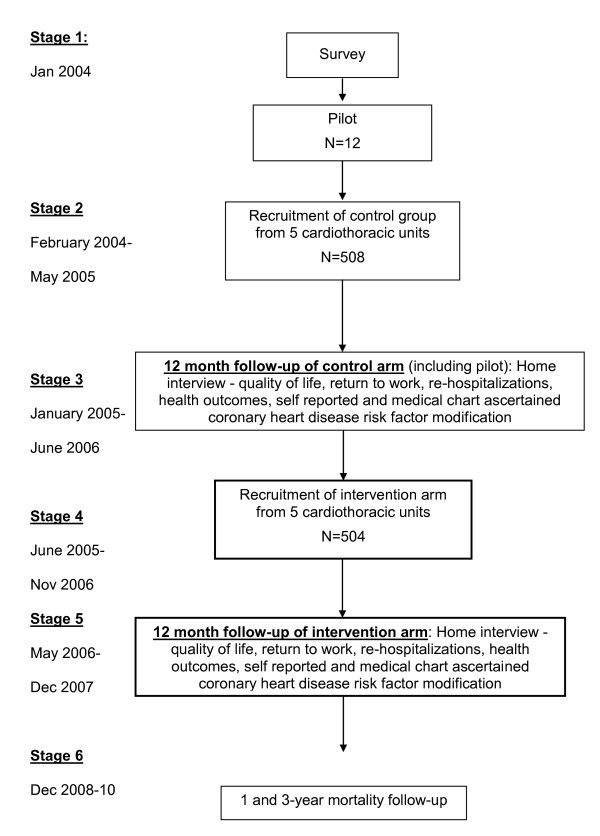
**Study Design**.

A controlled prospective study (Figure 1). The control arm comprised 520 patients who underwent CABG surgery between January 2004 and May 2005 in five medical centers across Israel. The intervention arm comprised 504 patients recruited from the same cardiothoracic departments between June 2005 and November 2006. Since the intervention included a seminar for the cardiothoracic unit staff, all patients in the control arm were recruited first. To avoid contamination, the implementation of our intervention and recruitment of the intervention arm took place upon completion of the control phase. In both arms, we approached consecutive patients scheduled for CABG surgery. Consenting patients were interviewed before surgery and one-year post surgery. The intervention group received oral and written explanation about CR programs and a telephone call after hospital discharge. In addition, the medical staff attended a one-hour seminar on CR and it was recommended that referral to CR be added to the patient letter of discharge. The control group received no additional treatment beyond usual care.

#### Selection of participating cardiothoracic units

We approached cardiothoracic units within medical centers located in different geographical regions of Israel, and of different affiliations (private, governmental, and health maintenance organization). The goal was to obtain a representative sample of the CABG patient population in Israel, regarding socioeconomic and ethnic characteristics, and health maintenance organization affiliation. We achieved collaboration with five medical centers. Agreement was initially sought from the medical director of each center, and subsequently from the head of each cardiothoracic ward and its head nurse. No directed action to increase cardiac rehabilitation rates had been taken in the participating cardiothoracic units prior to the study.

#### Calculation of sample size and study duration

We calculated sample size to enable detection of significant differences between patients in the intervention and control arms in the rate of participation in CR. We used estimates for these rates from published data on self-reported participation in CR in post-CABG patients [8]. The calculations for power, comparing the proportions [13] of participation in rehabilitation in the two arms, using a two-sided test with significance alpha = 0.05 and size of groups n1 = n2 = 430, and assuming that the proportional CR participation rate at baseline is 7%, yielded 90% power to detect a difference of 7% following intervention (that is, a rehabilitation rate of 14% in the intervention group). We assumed that up to 15% of the sample would fail to complete all the study items. Therefore, we increased the sample size accordingly to compensate for missing values. Thus, the targeted total for the intervention and control arms were 500 patients each, or 1000 in total.

According to data provided by the Israeli Ministry of Health, a total of 1596 CABG operations were carried out in the five units participating in the study during the year 2003. Assuming that these figures remained stable, and that exclusion criteria and refusal rate would reduce potential participation by 40%, we expected to reach our target sample of 1000 CABG patients (including pilot sample) within 15 months from study initiation. In reality, study was initiated in the five units at different times, as after the Institutional Review Board (IRB) approvals were obtained. As a result, the total duration of patient recruitment was longer than initially anticipated. The first 520 patients who enrolled in the study (from January 2004 to May 2005) composed the control arm. The following 504 patients, recruited between June 2005 and November 2006, composed the intervention arm (Figure 1).

We excluded from the study patients with severe co-morbidities for whom CR is contra-indicated (e.g. CHF stage IV); patients who were unsuitable for interview prior to their operation, i.e. patients undergoing emergency CABG operation; institutionalized patients; and patients with severe cognitive impairment (e.g. general stroke with severe disability); patients who did not understand any of the languages in which the study was conducted: Hebrew, English, Russian, and Arabic; and patients residing farther than 30 km from a rehabilitation center, due to the difficulty for these patients to commute to a CR facility.

**Figure 2:**
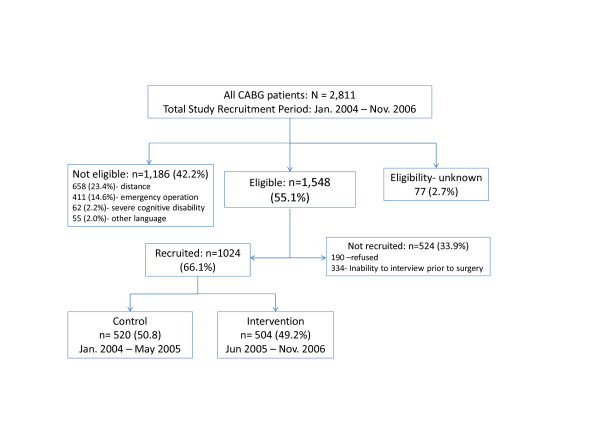
**Study population and study sample**.

For standardization, we interviewed all patients during their pre-surgery hospitalization. Patients who could not be interviewed prior to surgery (e.g. due to emergency operation) were not included since their mental or cognitive state could be affected by general anesthesia and CABG surgery (Figure 2).

### Procedure

#### Pilot study

We examined the pre-operative and post-operative questionnaires of the first 12 patients scheduled for CABG surgery in the cardiothoracic unit of the Hadassah Medical Center, Jerusalem, the first medical center in which we obtained IRB approval. We amended the interview protocol according to the results and feedback from this pilot study.

#### Study initiation, and recruitment of the control arm

Prior to recruitment, we presented the technical and logistic aspects of the study to the staff at each participating cardiothoracic unit. We recruited participants for the control arm from consecutive patients who were hospitalized while waiting for CABG surgery. Assisted by the chief nurse at each unit, a trained interviewer assessed each candidate's eligibility for participating in the study. After obtaining written informed consent, personal interviews were carried out, anthropometric measures obtained, and medical information e.g. results from blood tests, extracted from patients' medical chart.

#### One year follow up interview

Participants were approached again for a second interview one year after CABG surgery. Interviews were carried out by trained interviewers at participants' homes. The assessment included an interview and obtaining anthropometric measures and medical information from medical documents. Assessment at both time-points took approximately 60 minutes to complete. Patients were asked to prepare medical documentation for the interview. Those refusing a home interview were contacted by telephone; they provided information only regarding their attendance in a CR program (n = 118).

#### Intervention

We first asked physicians and nurses in each participating cardiothoracic unit to complete a short questionnaire to assess their level of knowledge regarding the content and provision of CR programs within the Israeli national healthcare system. This was followed by a one-hour seminar that included information for medical staff on the potential benefits of cardiac rehabilitation for patients and the provision of these programs within the healthcare system in Israel.

Recruitment and interviewing of the intervention arm was identical to that described for the control arm. In addition, patients in the intervention arm participated in an educational program that included 4 elements:

1. An individual 10-minute consulting session conducted during the hospital stay, prior to CABG surgery. We explained the benefits of cardiac rehabilitation, the content of such programs, criteria for patient eligibility for CR under the Israeli National Health Insurance Law, and the contact details of CR programs available in Israel and of the health maintenance organization (HMO) to which patients were affiliated. Sessions were delivered in Hebrew, English, Russian, and Arabic.

2. Pamphlets specially prepared for this study, in Hebrew and Russian, were distributed to patients at the end of the consultation session. The content was the same as that presented orally.

3. The director of each participating cardiothoracic ward was asked to issue each patient an explicit recommendation for participation in a CR program in their letter of discharge.

4. Two to three weeks after discharge from the hospital, each patient received a telephone call from a trained interviewer inquiring whether he or she intended to participate in a CR program. Patients were reminded of the importance of CR, and further encouraged to participate.

#### Mortality follow-up

The study included mortality follow-up at one-year and three-year post surgery. The study file will be merged with the Central Population Registry-Israel.

#### Validation of patient interviews

To verify and complete information on medications and comorbidities, as well as the respondents' self report on medical parameters, each interview was supplemented by information from patients' medical records: medical files, and medical documentation.

### Investigated variables

#### Main outcome (dependent) variables

Comparing intervention versus control groups: cardiac rehabilitation participation, assessed 1-year post surgery

Comparing participants and non-participants in cardiac rehabilitation: the following were recorded by a structured questionnaire administered twice, first at study entry (before surgery) and second at one-year follow up:

▪ Use of healthcare services during the previous 12 months, including the frequency of visits to a primary practitioner, the number of visits to a cardiologist, the number of re-admissions to the hospital or emergency department, and the reasons for these re-admissions.

▪ Lifestyle parameters associated with increased cardiovascular risk, including dietary habits, tobacco use, and employment status.

▪ Physical activity and change in exercise capacity; derived from a score on a standardized physical activity questionnaire. This questionnaire was validated against cardiorespiratory testing as a predictor of cardiovascular fitness [14].

▪ Mental health and quality of life, using the generic health-related quality of life SF-36 HRQL questionnaire [15,16].

▪ Quality of Life as assessed by the disease specific MacNew Heart Disease Health-related Quality of Life Questionnaire [17].

▪ Depression and Anxiety, measured using the Hospital Anxiety Depression Scale (HADS) questionnaire [18].

▪ Anthropometric and physiologic data: blood pressure (measured three times during the interview); BMI (calculated from weight and height measured during the interview), heart rate (measured during the interview), waist and hip circumference (measured during the interview); plasma glucose, hemoglobin, total cholesterol, HDL-cholesterol, LDL-cholesterol, triglycerides, WBC, ejection fraction (collected from medical records).

▪ All cause mortality (one-year and three-year follow-up) will be carried out by linking the study file with data from the Israeli Central Population Registry.

▪ For those who participated in a CR program: the type of program and its content, adherence to the program, co-payment and cost of service to the patient, satisfaction with the program, and barriers to adherence encountered by patients.

#### Independent variables and potential confounding factors

▪ Social and demographic parameters: age, gender, marital status, number of years of education, occupation, employment status, self-reported level of income (categorized in relation to the national average household income).

▪ Barriers to the use of CR services: poor health, work or financial constraints, lack of means of transportation.

▪ Health maintenance organization (HMO) affiliation and insurance coverage. We note that four national medical insurers/providers operate in Israel and a number of other medical insurance companies provide complementary services.

▪ Previous cardiovascular morbidity defined from the following question: "Did you undergo any previous cardiac procedures or cardiac related hospitalizations?"

▪ Co-morbidity defined from the questions: "Do you suffer from any other disease and/or handicap? If so, please specify the disease(s)/handicap and age at first occurrence"; "Have you been hospitalized in the last 12 months? If so, please specify the cause and month of each hospital admission."

▪ Medical treatment information: medications prescribed. This information is complementary to the information on self-reported conditions associated with cardiovascular disease (i.e., hypertension, diabetes mellitus, and hyperlipidemia) and other chronic diseases. Data was supplemented by information from medical files at hospitals.

▪ Severity of coronary disease: % ejection fraction (available for about half the patients), number of occluded vessels prior to CABG.

### Study population

#### Characteristics of participating patients

Of the 2811 CABG patients who underwent CABG surgery at the five participating cardiothoracic wards during the study period, 1024 (36%) were recruited to our study. Table 1 compares the demographic characteristics of the participants to the 2811 patients who underwent CABG surgery. As in the total post-CABG population, the proportion of women in our sample was approximately 24%; their mean age was about 5 years older than that of men.

**Table 1:** Demographic characteristics of the post-CABG patients who participated in the study

Characteristic	All CABG patients n (%)	Participating CABG patients n (%)
Total number	2811 (100)	1024 (100)
Control arm	1401 (49.8)	520 (50.8)
Intervention arm	1410 (50.2)	504 (49.2)

% women	671 (23.9)	244 (23.8)

Age (mean and SD) [range]		
All	65.3+10.7 [30-92]	65.3+10.7 [37-91]
Women	69.7+10.2 [33-91]	70.0+9.2 [40-87]
Men	63.6+10.4 [30-92]	64.6+10.4 [37-91]

Ethnicity % **		
Jews - veteran Israeli citizen	1902 (64.1)	757 (73.9)
Jews - recent immigrants	589 (20.9)	216 (21.1)
Arabs	280 (13.0)	47 (4.6)
Other	40 (1.4)	4 (0.4)

Medical center		
Tel Aviv	777 (27.6)	363 (35.5)
Hadassah	497 (17.7)	146 (14.3)
Carmel	602 (21.4)	175 (17.1)
Asuta	351 (12.5)	154 (15.0)
Ramat Marpe	584 (20.8)	186 (18.2)

** Jews - veteran Israeli citizen are patients born in Israel or those who immigrated to Israel prior to 1989.

Jews - recent immigrants are patients who immigrated to Israel from the former Soviet Union since 1989, when about 750,000 Jews came to Israel during a short period of time

Arabs - Israeli Arab citizens

Other Israeli citizens not belonging to any of the above definitions.

Although veteran Israel citizens compose 64% of the population undergoing CABG surgery in Israel, they composed 74% of our sample. In contrast, the Arab population comprised 13% of the CABG population but only 4.6% of the study sample. This resulted mainly from the fact that the majority of Arabs reside in remote places, more than 30 kilometers from a cardiac rehabilitation center. Of those eligible to participate in the study, logistic difficulties, i.e. unavailability of an Arabic speaking interviewer, were the main causes for non-participation. There was no over-representation in refusal to participate among Arabs or any other ethnic group.

Differences in the five medical centers between the total number of patients undergoing CABG surgery and the number of participants in the study are also due, at least in part, to availability of interviewers. Patients who arrived at the hospital in the evening and underwent surgery early in the next morning were often not available for pre-surgical interviews.

#### Information regarding non-participants

To assess a possible selection bias, we collected information on all the patients who were not included in the study. A short semi-structured form including parameters such as age, sex, country of birth, ethnicity, medical center, and the reason for exclusion was filled.

### Data collection and analysis

#### Data collection, coding, and quality control aspects

To ensure standardization, periodic training was provided for interviewers. To achieve accuracy, data was collected in structured forms. Information was coded by a trained nurse who also carried out data entry and data quality assurance. All study questionnaires were reviewed for inaccuracies and inconsistent data entry. Quality assurance of personal interviews, as well as medical information, was carried out by the research coordinator. The study team convened at monthly meetings to review data outputs and discuss relevant issues, to standardize interviewing techniques, and to address potential problems.

#### Methods for statistical analysis

The analysis will constitute two parts according to the main study hypotheses: the first analysis will compare the intervention and the control arms to assess the impact of the intervention on the rate of CR uptake, and to assess the role of mediating factors. The second analysis will compare participants in CR programs and patients who did not participate in CR programs to evaluate the impact of CR on medical and psychosocial outcomes.

Univariate associations between the outcome measures in the comparison groups, and by various independent covariates, will be explored using the χ^2^-test, t-tests, and non-parametric procedures. To test for differences between two populations regarding continuous variables, we will use the unpaired t-test for normally distributed variables and the Wilcoxon rank sum test otherwise. To test for differences between two groups for various outcomes, adjusting for risk factors and potential confounders, we will use the generalized linear model for continuous outcome variables and the logistic regression model for dichotomous variables.

The medical center where the operation took place will be examined as a confounder to account for differences in the populations treated at each cardiothoracic unit, in the medical approach implemented, and in possible effects on outcomes.

For investigation of the second goal, we will compare those who did and did not participate in a CR program using the same tests as for comparison of control and intervention groups. We are aware that participation or non participation in CR depends on other parameters and can not be considered as randomly assigned. Therefore, we will apply the propensity score approach. First we will build a model for the probability of participation in CR (i.e. the propensity score) that will be based on a set of demographic and medical variables. This propensity score will be used in the regression analysis of the effect of participation in CR on various outcomes.

### Ethics

The study was approved by the IRB of the Sheba Medical Center and of each participating medical center. An informed signed consent was obtained from all participants prior to the first interview. Data confidentiality has been maintained in accordance with current regulations.

The study is registered in ClinicalTrials.gov as NCT00356863.

## Discussion

Since our planning and launching of this study, considerable evidence has accumulated about the benefits of cardiac rehabilitation (CR) [19], as well as about low participation rates in CR programs [20-22]. Interventions have focused on means of increasing referral rates. Referral strategies have been categorized as 'automatic' (by electronic health records or systematic discharge); 'liaison' (discussions with health-care providers), and 'other' (such as written material and phone calls) [23]. A recent review concluded that automatic referral orders attain the highest referral rates, and a combination of automatic and liaison methods attain the highest rates of CR enrollment [24]. In a recent Canadian study, automatic referral and liaison methods, alone, and even more so when combined, considerably increased CR participation [23]. Increasing physician awareness of the benefits of CR has also been shown to increase patient participation [25].

Guidelines issued in 2007 by the American Association of Cardiovascular and Pulmonary Rehabilitation (AACVPR), the American College of Cardiology (ACC), and the American Heart Association (AHA) recommend automatic referral to cardiac rehabilitation for every eligible patient [26]. In April 2011, the Canadian Association of Cardiac Rehabilitation (CACR) and the Canadian Cardiovascular Society (CCS) issued a joint position statement recommending a combined approach to increasing CR participation, consisting of a checklist or electronic referral and talking with patients [23].

### Study strengths

This large prospective study was carried out in four languages in five medical centers across Israel. We designed a simple and low cost intervention that combined the primary elements that have since been recommended for increasing CR referral, as delineated above: automatic referral; liaison; written material and phone calls to patients; and increasing awareness of physicians. We conducted a comprehensive assessment, with a relatively long follow-up, of clinical, psychosocial and sociodemographic patient outcomes, and collected considerable data of the CR programs attended.

### Methodological considerations

We made every effort to include all eligible patients in the study, and collected data on eligible patients who were not recruited. We note that there were no differences in mean age or gender distribution between the participating and non-participating CABG patients (Table 1). The main reasons for not participating in the study were refusal and logistical problems, mostly due to the inability to coordinate an interview before CABG surgery.

Unlike many studies on intervention programs that use referral rate or rate of enrollment as their end-point, we assessed actual participation in CR programs.

The sequential recruiting of the control and interventional arms ensures identical settings. However, though no CR interventions were conducted during recruitment of the control arm, we cannot ignore the possibility that awareness of the medical staff to the benefit of CR may have increased during that period by means other than by our intervention.

### Expected outcomes and contribution

We expect our intervention to increase the rate of CR participation in the intervention arm relative to the control arm (usual care). We also anticipate that CABG patients who participate in CR programs will have better health and quality of life outcomes compared to patients who do not participate in such programs.

## Competing interests

The authors declare that they have no competing interests.

## Authors' contributions

RD conceived the study, and led its design and coordination. She also participated in data collection, data analysis, and helped to draft the manuscript. GG participated in study coordination, data collection and analysis, and helped to draft the manuscript. AZ participated in quality control, establishment of the data file, and statistical analysis. IN participated in the design of the study and performed the statistical analysis. UG and YD participated in the conception of the study, and in its design. All authors read and approved the final manuscript.

## Pre-publication history

The pre-publication history for this paper can be accessed here:

http://www.biomedcentral.com/1471-2261/11/60/prepub
